# Spatial single cell analysis of tumor microenvironment remodeling pattern in primary central nervous system lymphoma

**DOI:** 10.1038/s41375-023-01908-x

**Published:** 2023-04-29

**Authors:** Yuan Xia, Tao Sun, Guosheng Li, Mingying Li, Dongmei Wang, Xiuhua Su, Jingjing Ye, Chunyan Ji

**Affiliations:** 1 Department of Hematology, Qilu Hospital of Shandong University, Cheeloo College of Medicine, Shandong University, Jinan, 250012 PR China; 2 Shandong Key Laboratory of Immunohematology, Qilu Hospital of Shandong University, Cheeloo College of Medicine, Shandong University, Jinan, 250012 PR China

**Keywords:** Cancer microenvironment, Immunoediting

## Abstract

To determine the overall tumor microenvironment (TME), characteristics, and transition mechanisms in primary central nervous system lymphoma (PCNSL), we performed spatial transcriptomics and matched the corresponding single-cell sequencing data of PCNSL patients. We found that tumor cells may achieve a “TME remodeling pattern” through an “immune pressure-sensing model”, in which they could choose to reshape the TME into a barrier environment or a cold environment according to the immune pressure. A key *FKBP5*^*+*^ tumor subgroup was found to be responsible for pushing tumors into the barrier environment, which provides a possible way to evaluate the stage of PCNSL. The specific mechanism of the TME remodeling pattern and the key molecules of the immune pressure-sensing model were identified through the spatial communication analysis. Finally, we discovered the spatial and temporal distributions and variation characteristics of immune checkpoint molecules and CAR-T target molecules in immunotherapy. These data clarified the TME remodeling pattern of PCNSL, provided a reference for its immunotherapy, and provided suggestions for the TME remodeling mechanism of other cancers.

## Introduction

Tumor microenvironment (TME) plays an important role in tumorigenesis, development, metastasis and drug sensitivity [1]. Knowledge of tumor-immune system interactions has provided a basis for rational guidance on patient stratification and surgical strategies, as well as a more comprehensive understanding of possible intervention points and the causes of treatment failure [2–4]. Based on the tumor-immune system interaction pattern, a more comprehensive classification method classifies the TME into four main types — “hot”, “invasive margin excluded (IME)”, “invasive margin immunosuppressed (IMS)”, and “cold” [5]. It has been reported that in some tumors, such as colon cancer and melanoma, the hotter the TME is, the better the patient survival [6, 7]. However, the high heterogeneity of the TME remains a key barrier to the understanding and treatment of cancer, suggesting that mapping the composition and functional status of the tumor-immune system in the TME is of great significance. Many efforts have uncovered some variations in TME heterogeneity and concurrence that accompany malignancy in different cancer types. However, most studies have focused on the composition of TME and largely ignored its spatial distribution specificity [8]. Therefore, a detailed understanding of the progression of phenotypic changes that occur during the transition of oncogenic status, as well as the molecular drivers of this transition, remains to be explored.

Primary central nervous system lymphoma (PCNSL), a highly invasive non-Hodgkin lymphoma, occurs in the central nervous system (CNS), including the brain, spine, cerebrospinal fluid (CSF) and eye [9, 10]. Patient survival is generally low compared to lymphomas outside the CNS, and PCNSL that fails first-line treatment has a poor prognosis, which makes the treatment of this disease challenging [11]. Although a previous study investigated the cell composition and gene expression patterns of PCNSL at single-cell level [12], the different space positions of the cells in one TME were still in a mixed state with the process of sample preparation and sequencing, thus missing the spatial orientation. Therefore, combining new methods to clarify the spatial and temporal development patterns of the TME and the key occurrence mechanism is of great importance. At the same time, current research suggests that blood-brain barrier permeability increases after tumor occurrence, and the TME in the brain has cell types similar to those outside the brain [13]. Therefore, our study on PCNSL is likely to provide useful clues for the TME study of other solid tumors as well.

Here, 14964 single-cell transcriptomes of PCNSL were combined with spatial transcriptome analyses of hot, IME, IMS, and cold TMEs. The TMEs were characterized. In brief, the main cell types in the TME of PCNSL patients were identified by single cell sequencing, and were mapped to the corresponding spatial locations in 4 TMEs. Through integrative analysis, we defined and annotated tumor cell subpopulations according to their spatial distribution and functional characteristics and found that various tumor cells work together to reshape the TME into a barrier or cold environment through an “immune pressure-sensing model”, thus realizing the “TME remodeling pattern”. A key *FKBP5*^*+*^ tumor subgroup was found to be closely associated with TME remodeling, which provides a possible method for assessing brain tumor staging. The spatial communication mode between tumor cells and immune cells in each TME was also identified, thus refining the specific mechanism of the TME remodeling pattern and identifying the key molecules of the immune pressure-sensing model. Corresponding treatment suggestions were proposed according to the spatial characteristics of each TME in current PCNSL immunotherapy. These results reveal the spatial heterogeneity of PCNSL, highlight the localization and status of cell types and potential intercellular signals in the TME, and provide resources for further research on TMEs and updating immunotherapy methods.

## Materials and methods

### Human patient samples

All patient tissue sections of PCNSL used in the study were obtained from the Pathology Department of Qilu Hospital, and all patients signed informed consent forms before sampling. After screening qualified patient sections, pathologists scored the sections according to the immune scoring system, and finally screened out 4 tissue sections that met the main types of TMEs.

### Spatial transcriptomics

#### Slide preparation

**Fig. 1:**
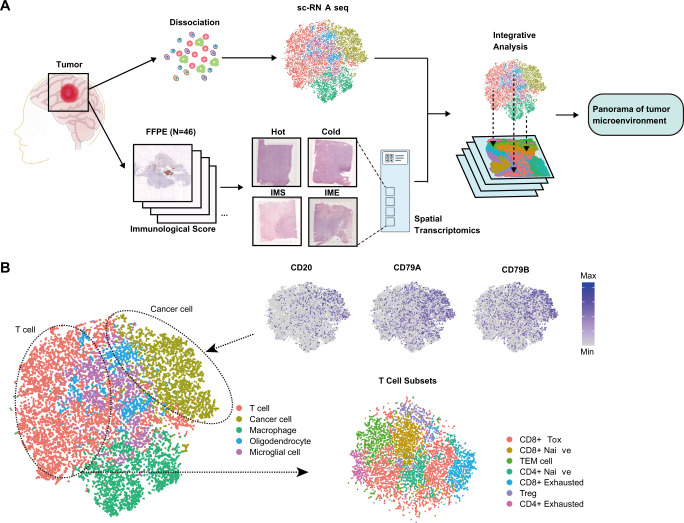
Landscape of the PCNSL TME. **A** Workflow of patient sample screening, single-cell sequencing, spatial transcriptomics and combined analysis. **B** Main cell types and tumor cell marker validation and T-cell subsets in TME of PCNSL patients.

Spatial transcriptomics slices print 4 identical 6.5 × 6.5 mm capture regions, each with 5000 cluster spots (10X Genomics). The spots, with a diameter of 55 μm, the core spacing between adjacent spots is about 100 μm. Primer sequences on each spot included Read1 sequencing primer sequence, 16nt Spatial Barcode sequence, 12nt UMI sequence, and 30nt PolyT primer sequence. A single chip can be used to detect four different tissue section samples at the same time, but the permeability conditions of the samples need to be optimized and determined respectively. The number of captured cells corresponding to a single spot region is in the range of 1–10 (according to the sizes of cells), so it can be considered that the detection level of spatial transcriptome is close to the single-cell level (Fig. 1A).

#### Section fixation, staining and imaging

Sections were incubated at 37 °C for 1 min, fixed with 4% formaldehyde (Sigma-Aldrich) in PBS for 10 min, and then washed with 1 × PBS for 3 times. For staining, the sections were incubated in hematoxylin (Solarbio, CHN) for 4 min, bluing buffer (Solarbio, CHN) for 30 s, and eosin (Solarbio, CHN) for 30 s. After each staining step, sections were washed with 1 × PBS. After air drying, the slides were scanned using a Zeiss platform (Carl Zeiss AG, Germany) at 20 × magnification of brightness field images.

#### Tissue permeabilization

The slides were inserted into the slide box, and the tissue sections were separated into separate reaction chambers. For prepermeability, the sections were incubated at 37 °C with 0.5 U/mL collagenase (ThermoFisher) and 0.2 mg/mL BSA (ThermoFisher) for 20 min in HBSS buffer (ThermoFisher). The wells were washed with 0.1 × SSC (Sigma‒Aldrich), dissolved with 0.1% pepsin (Sigma‒Aldrich) in 0.1 m HCL (Sigma‒Aldrich) and permeated at 37 °C for 10 min. After incubation, the pepsin solution was removed and washed with 0.1 × SSC.

#### Library preparation and quantification

After reverse transcription, Qubit 3.0 was used for preliminary quantification, and the library was diluted to 1 ng/ul. Then, Agilent 2100 was used to detect the insert size of the library. After the insert size met the expectation, Q-PCR was performed using StepOnePlus Real-Time PCR System to accurately quantify the effective concentration of the library (the effective concentration of the library was required to be > 10 nM) and ensure the quality of the library. Qualified libraries were sequenced using the Illumina platform.

#### Spatial transcriptomics raw data processing

Illumina platform sequencing was used to obtain Raw off-machine sequences (Raw reads), and data interception was performed to obtain sequences to be analyzed (Clean reads). All subsequent analyses were based on Clean reads with GRCh38 V86 genome compilation as a reference and the corresponding GENCODE annotation file (version 25). The count matrix was screened, only protein-coding genes, long noncoding genes and antisense genes were retained, and the Ensembl ID was replaced with HGNC symbols.

#### scRNA-seq Data processing

The specific parameters of quantification and cell screening were referred to previously published literature [12, 14]. In brief, the gene barcode count matrix was analyzed using the Seurat R software package (version 4.0.2). Cells with > 200 genes and < 10% mitochondrial gene profiles were screened from downstream analysis. After the samples were converted to Seurat, the combined Seurat objects were normalized and scaled by regression of UMI count and mitochondrial gene percentage. In terms of dimension reduction, the FindVariableGenes function was used to identify the most variable genes. PCA was then used for dimensionality reduction, and the TSNE graph was generated by the RunTSNE Seurat function (Seurat version 3.1.3).

#### Cell type annotation

After cell clustering, the FindAllMarkers function of Seurat was used to generate DEGs (logFC > 0.25 and P < 0.05) for each cluster in scRNA-seq data. We compared all DEGs of each cluster with the CellMarker database to get the corresponding marker genes of each cluster, and annotated the single cell data according to the obtained specific marker genes. The cell annotation method for spatial omics sequencing data was referred to the previously published literature [14]. By calculating the DEGs for each cell type in single-cell data using FindAllMarkers function, the top 30 DEGs of each cell type were used to score each spot for spatial omics sequencing data after inspection and clustering, and the cell type with the highest score for each cluster was used for annotation.

#### Spatial transcriptomics data processing

Based on the gene expression matrix in spots obtained from 10X Genomics Visium, PCA was used to reduce the dimensionality of expression data, and then cluster analysis was performed using PCA dimensionality reduction data. The SCTransform function was used to normalize each locus, and the number of copies and genes at each locus was regressed. The first 20 ICs were reduced and clustered using independent component analysis (ICA) at a resolution of 0.8.

#### Pathway analysis

The function FindMarker provided by Seurat was used to identify DEGs (logFC > 0.25 and *P* < 0.05) in each cluster for spatial transcriptomics data and scRNA-seq data. ClusterProfiler was used for GO enrichment analysis of all DEGs in each cluster. As for spatial transcriptomics data, after getting the top 10 pathways of each cluster, we searched the upper-level pathway of each pathway one by one through the GO official website (http://geneontology.org) and group the pathways with the same uppermost pathway name together. GSVA was carried out with the GSVA package.

#### Trajectory analysis

Monocle2 R package (version 2.20.0) were used for the trajectory analysis. we selected the top 2000 significant DEGs among all cell types as the ordering genes. Dimensionality reduction and trajectory construction were performed on the selected genes with default methods and parameters. Tumor cell subsets in the four TMEs were first used separately for trajectory analysis, and then all tumor cells in the four TMEs were extracted for co-trajectory analysis. BEAM analysis was used to get the expression patterns in branches during development. Gene Switch analysis was performed using GeneSwitches R package (version 0.1.0) that can process trajectories to identify genes that act as switches between cellular states [15]. Based our results from co-trajectory analysis, GeneSwitches first binarizes each gene in each cell to facilitate the identification of switching events while the pseudotime of each cell providing the independent variable. The probability of gene-expression throughout pseudotime was calculated and the quality of fit was estimated using Pseudo R^2^. The activated switching genes with pseudotime (R^2^ > 0) were defined as upregulation genes, otherwise down-regulated genes. The higher the absolute value of R^2^, the closer the relationship between switching genes and the trajectory process.

#### Cell chat analysis

Cell Chat Analysis was performed using CellChat R package (version 1.5.0) [16]. Briefly, we followed the official workflow and default parameter settings to load the four TME data separately into CellChat after quality inspection and normalization. The built-in CellChatDB.human database was used as a reference for screening receptor-ligand interactions. The potential ligand-receptor interactions between infected and non-infected cells and potential pathways were calculated using computeCommunProb, computeCommunProbPathway and aggregateNet functions with standard parameters.

#### Tumor staging analysis

We used the Gene Expression Profiling Interactive Analysis (GEPIA) database (http://gepia.cancer-pku.cn) and the TCGA data contained therein to analyze the staging of the tumors [17]. The GEPIA database is an online platform that uses a common processing technique to examine RNA sequencing expression data from the TCGA and the Genotype-Tissue Expression (GTEx) projects. GEPIA also has interactive features including profiling based on pathological stages, and it has incorporated statistical methods to process the data.

## Results

### Landscape of the PCNSL TME

The complete TME status of the sample were took into account when scoring each sample. For example, the complete TME of hot tumor was that a large number of T cells were widely distributed in both the interior of the tumor and the invasive margin. In cold tumors, however, there are very few T cells, either inside or around the invasive margin of the tumor. The complete TME of IMS is that there are few T cells inside the tumor, but a few T cells are distributed around the invasive margin. IME tumor also had very few T cells inside, but a large accumulation of T cells around the invasive margin (Supplementary Fig. S1A). Finally, 4 pathological sections that could best reflect 4 representative TMEs were selected from 46 patients with PCNSL for spatial transcriptome analysis. In order to solve the problem of insufficient spatial transcriptome resolution, we matched the single cell transcriptome data of PCNSL to annotate the main cell types in the spatial transcriptome and conduct integrative analysis. (Fig. 1A). We processed the single cell sequencing data according to the parameters mainly from the source literature [12], and some parameters missing in the source literature from other literature [14]. 14964 cells were observed from the TME after quality control and screening. Then, we manually annotated the cell clusters into 5 different cell types with distinct gene expression patterns, including T cells, macrophages, microglial cells, oligodendrocytes and cancer cells. (Fig. 1B and Supplementary Fig. S1B). Three representative markers, *CD20*, *CD79A* and *CD79B*, were used to test the accuracy of the tumor cell population. Considering the diversity and importance of T cells in the TME, T cells were extracted and manually annotated into 7 clusters according to the distinct gene expression patterns of each cluster, including CD4^+^ naive, Treg, CD4^+^ exhausted, effect memory T-cell (TEM), CD8^+^ naive, CD8^+^ toxic T-cell (TOX) and CD8^+^ exhausted cells (Fig. 1B and Supplementary Fig. S1C).

### Spatial morphology and heterogeneity of the PCNSL TME

**Fig. 2:**
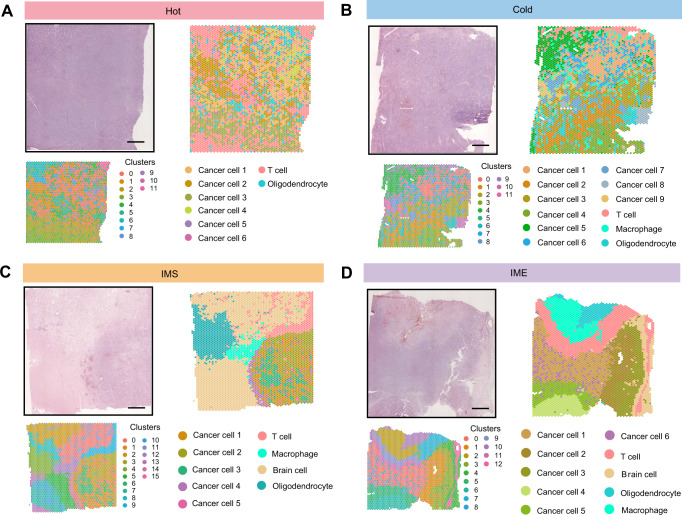
Spatial morphology and heterogeneity of the PCNSL TME. **A** Hematoxylin and eosin (H&E) staining of the “hot” TME region with the corresponding unbiased clustering of ST spots and the spatial distribution of each cell subpopulation after annotation in the “hot” TME. Scale bar, 1 mm. **B** Hematoxylin and eosin (H&E) staining of the “cold” TME region with the corresponding unbiased clustering of ST spots and the spatial distribution of each cell subpopulation after annotation in the “cold” TME. Scale bar, 1 mm. **C** Hematoxylin and eosin (H&E) staining of the “IMS” TME region with the corresponding unbiased clustering of ST spots and the spatial distribution of each cell subpopulation after annotation in the “IMS” TME. Scale bar, 1 mm. **D** Hematoxylin and eosin (H&E) staining of the “IME” TME region with the corresponding unbiased clustering of ST spots and the spatial distribution of each cell subpopulation after annotation in the “IME” TME. Scale bar, 1 mm. See also Supplementary Fig. S1 and Fig. S2.

Based on the immune score, four typical immune microenvironments were selected for spatial sequencing and formed different clusters at the spatial level (Fig. 2A–D). A same method as the previous report was performed to annotate these clusters [14]. By calculating the gene expression profile of each cell type in PCSNL single-cell data (Supplementary Table 1), we intercepted the top 30 genes of each cell type to score each spot in the spatial transcriptome, and the cell type with the highest score for each cluster was annotated. Through the score of T cells and tumor cells, we confirmed that our selected slices indeed fit the characteristics of hot immune microenvironment, which is found with the most infiltrated immune cells (Fig. 2A and Supplementary Fig. S2A, Fig. S2C). We verified the accuracy of our definition by using the expression of specific markers and confirmed our annotations (Supplementary Fig. 2B). In addition, considering the heterogeneity of tumor cells in TME, different clustering states and numbers of tumor cells were maintained, and tumor cells were annotated into different clusters (Fig. 2A). In the cold environment, only a few T cells infiltrated, and the tumor cells still formed distinct clusters in space level (Fig. 2B and Supplementary Fig. S2A, Fig. S2D, E). For the other two intermediate states, the IMS tumor environment had only a small number of T cells and macrophages gathered in the boundary of the tumor area (Fig. 2C and Supplementary Fig. S2A, Fig. S2F, G), at the same time, the tumor cells appeared to have more obvious spatial heterogeneity, the outer and inner parts of the tumor formed different clusters, which may indicate different divisions of labor (Fig. 2C). The IME tumor environment was defined as a state that had a large number of immune cells in the invasive margin (Fig. 2D and Supplementary Fig. S2A, Fig. S2H, I). The tumor heterogeneity and spatial distribution of specificity were amplified in IME tumors. Tumor cells moved from the inside out to form an “onion cascading structure”, and each layer seemed to have a different status (Fig. 2D). All the results indicated that the tumor cells were inhomogeneous, they formed their special spatial distributions, and may have their respective division of labor.

### Definition and notation of PCNSL cell subpopulations

**Fig. 3:**
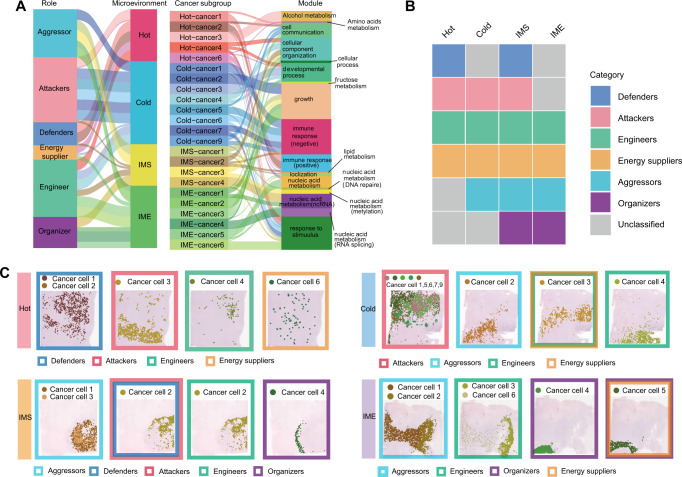
Definition and notation of PCNSL cell subpopulations. **A** GO pathways and role definitions for each tumor cell subpopulation of the four TMEs. **B** Tumor cell role types for each TME. **C** The subpopulations of various role types of tumor cells in each TME and their spatial distribution. See also Supplementary Fig. S3, Supplementary Table S1, S2, and S3.

To further explore the specific function of each tumor cluster, we performed the differentially expressed gene (DEG) analysis for each cell cluster using annotated spatial transcriptome data. Gene Ontology (GO) enrichment analysis was performed for all DEGs of each cancer cell cluster, and the top 10 GO terms were identified to reflect the functions of each cluster of cancer cells (Supplementary Table 2). We searched the upper-level pathways of each term one by one through the GO official website, and used the uppermost parent pathway to summarize the pathways and defined each cluster of cancer cells according to a similar function, which refers to their role in the microenvironment (Fig. 3A, B, Supplementary Fig. S3A and Supplementary Table 3). To determine whether the tumor was in a passive defensive or aggressive state relative to the immune cells in the microenvironment, we performed gene set variation analysis (GSVA) for immune regulation pathways in each microenvironment. It was found that most of the tumor cells in hot tumors were in a state of passive defense (Supplementary Fig. 3B), while the tumor cells in cold tumors basically lost the supervision of immune cells and were in a state of negative immune regulation. For IMS tumors, the two states coexisted. IME tumors were also in an immunosuppressive state. Therefore, we defined passive defense tumor cells in hot tumors as “defenders”, mainly including cancer clusters 1 and 2. From the spatial position, they were mainly distributed in the upper part of the section. Since tumor 3 had a very strong sterol metabolism function to fight against immune cells, we defined it as an “attacker”. Cancer clusters 4 and 6 were scattered in the whole space and were responsible for auxiliary work, such as cell movement and energy supply, so they were named “engineers” and “energy suppliers” (Fig. 3C).

In cold tumors, there were no cancer cells in the immune defense state, and most cancer cell clusters, such as clusters 1, 2, 6, 7, and 9, showed a strong negative immune regulation ability (Fig. 3C). They also showed strong consistency in function, mainly distributed in the upper part of the section. It is worth noting that cold tumors had a cluster of highly proliferative cancer cells (cluster 2), so these tumor cells were named “aggressors”. In addition, auxiliary cancer clusters 3 and 4 existed in the intersection zone of attackers and aggressors.

The state of cancer cells in IMS tumors had similarities with the first two states but also had its own differences. Cancer cluster 2 in the IMS tumors, in direct contact with T cells, showed both positive and negative immune regulation states and seemed to be in the transition stage of fighting T cells, while cancer clusters 1 and 3, relatively close to the interior, showed strong proliferative characteristics. It was noteworthy that a cluster of cells in IMS tumors were different from the cancer populations in cold and hot tumors. Since the main function of cancer cluster 4 in IMS tumors was metabolic reprogramming of ncRNA, it was named “organizers” (Fig. 3C). A same cluster was also found in the IME tumors, and it seemed to inhabit a core of space position, which superimposed over the internal areas of the tumor. With cancer cluster 4 as the center extending outwards, cancer cluster 5 surrounding it had a similar function to cancer cluster 4 and had a strong ATP metabolism ability. In addition, there were proliferating cells in cancer clusters 1 and 2, tumors 3 and 6 contained helper cells (Fig. 3C). In addition, we also extracted tumor cells from the scRNA-seq data of PCNSL patients, and calculated the DEGs of each cluster of cells after clustering. Then all the DEGs of each cluster were successively analyzed by GO enrichment analysis to verify the presence of each role of tumor subsets in our spatial transcriptome data (Supplementary Fig. 3C). Our results further clarified the spatial heterogeneity of tumors by completely annotating each cancer cluster according to their different division of labor in spatial dimensions.

### TME remodeling pattern and immune pressure-sensing model

**Fig. 4:**
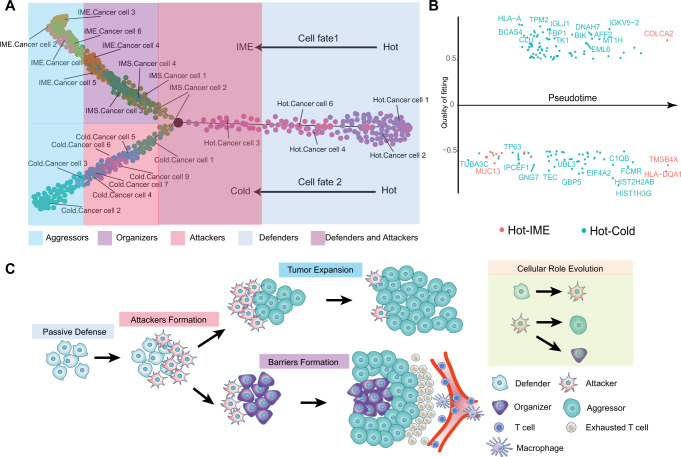
TME remodeling pattern and immune pressure-sensing model. **A** Formation trajectory of the TME remodeling pattern. **B** Gene switch analysis of two cell fates of the TME remodeling pattern and scatter plot of key regulatory genes. **C** Schematic diagram of the immune pressure-sensing model. See also Supplementary Fig. S4 and Fig. S5.

After functional annotation of PCNSL tumor subsets, we found that some cancer clusters seemed to have a strong correlation and similarity, although they were in different TMEs. Some potential developmental correlations are emerging. So we explored the developmental trajectories of tumor clusters for each TME in detail. In hot tumors, defenders developed relatively early, attackers gradually developed against immune cells, and the auxiliary subgroups were involved in the whole development process (Supplementary Fig. 4A). In cold tumors, the attackers were found at an earlier stage, and with the help of the auxiliary subgroup, the aggressors developed thereafter. Cold tumors seemed to be a continuation of hot tumors, as if the cold environment was a later stage in the development of the tumor (Supplementary Fig. 4B). Compared with the other two transitional states, IMS tumors and IME tumors, there was no clear definition of their specific developmental stage in previous reports. Our studies found that clusters of cancer cells in IMS tumors that were in direct contact with T cells were in the earlier stage of the development axis, and then aggressors and organizers were derived, which developed in a spatial location far away from T cells (Supplementary Fig. 4C). IME tumors starts with organizers in IMS tumors and derive aggressors outwards (Supplementary Fig. 4D). Considering the crosslinks existed between some cancer clusters in different TMEs, we extracted all tumor cells from the four TMEs for combined analysis and obtained an overall view of the development trajectory. The hot tumor, as the starting point of development, had two different cell fates at the late stage, one fates to the cold tumor and the other fates to the IME tumor, while the IMS tumor was the transition state. We call this evolutionary form of tumor cells the “TME remodeling pattern” (Fig. 4A). We conducted Gene Switch analysis and obtained the key switch genes for hot tumor to cold tumor development, and for IME tumor development (Fig. 4B and Supplementary Fig. S5B, C). To provide some hints for how to transform tumors from cold into hot, which is what researchers are concerned about, branched expression analysis modeling (BEAM) analysis was performed on the key nodes of hot tumors developing in two directions, and the top 50 key genes at the critical juncture of tumor developmental transition were obtained (Supplementary Fig. S5A). In this way, we were able to link different tumor cell subpopulations in the 4 microenvironments together and understand their interrelationships and developmental axes (Fig. 4C). After experiencing the challenge of immune cells, defenders derived attackers, and attackers generated aggressors to expand the tumor when the pressure of immune cells was low, while organizers were derived to organize the tumor cells to construct the barrier environment in the area with high pressure of immune cells, such as the IME environment with a large distribution of blood vessels (Fig. 2D and Supplementary Fig. 5D). We call this form of immune pressure on the selective fate of tumor cells the “Immune pressure-sensing model”. As seen from the role of the transformation process of tumor subsets, organizers were crucial to the generation of barrier effects, so we conducted an in-depth study on the key marker of organizers.

### The *FKBP5*^*+*^ subpopulation promotes the formation of the TME remodeling pattern

**Fig. 5:**
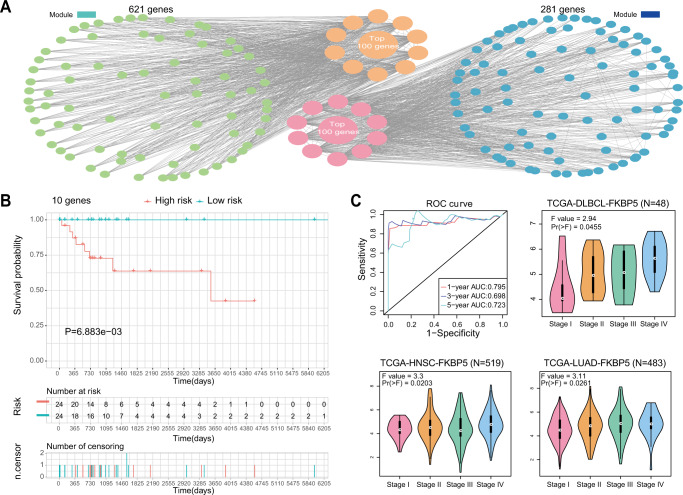
*FKBP5*^+^ subpopulation promotes the formation of the TME remodeling pattern. **A** The top 100 hub genes of each module screened by Cytoscape. **B** Survival analysis of the 10-gene signature-based risk score in the TCGA cohort. **C** The predictive capacity of the risk score for the 1- year, 3- year and 5- year survival rates of patients in the TCGA cohort (Upper left). Stage plot of *FKBP5* in lymphoid neoplasm diffuse large B-cell lymphoma patients (Upper right). Stage plot of *FKBP5* in head and neck squamous cell carcinoma patients (Lower left). Stage plot of *FKBP5* in lung adenocarcinoma patients (Lower right). See also Supplementary Fig. S6 and Supplementary Table S4, S5 and S6.

To identify the key marker of organizers and obtain clues for breaking the barrier environment to guide the selection of PCNSL treatment plan and the development of new drugs, we extracted organizers from the related TME and conducted weighted gene coexpression network analysis (WGCNA). According to the gene expression characteristics, organizers were divided into the blue and green modules (Supplementary Fig. S6A). According to the most critical pathways of organizers, it can be found that the green module mainly corresponds to mRNA processing ability, while the blue module mainly corresponds to ncRNA metabolism and RNA splicing ability (Supplementary Fig. S6B). We used Cytoscape to extract the top 100 hub genes in each module (Fig. 5A and Supplementary Table 4). These genes were combined with the clinical information of diffuse large B lymphoma patients for Lasso regression and Cox multivariate regression, and 10 genes closely related to the survival of patients were finally screened out (Fig. 5B, C and Supplementary Tables 5, 6). Considering that the formation process of organizers is associated with the progression of the tumor, we carried out further gene screening in patients with tumor stage information using the Gene Expression Profiling Interactive Analysis (GEPIA) database and the TCGA data contained therein and found that only *FKBP5* was closely related to the patient’s tumor progression, which was also associated with the progression of two other solid tumors (Fig. 5C). Firstly, based on the reality of no sufficient standard PCNSL patient data, associated tumor types which could be used to evaluate and compare the staging of patients with PCNSL were selected. For example, for DLBCL (Diffuse large B-cell lymphoma), 90% of patients with PCNSL belong to DLBCL, and it was chosen as the first option. For HNSC (Head and neck squamous cell carcinoma), as the location of this cancer is similar to PCNSL, it was also chosen. In addition, considering the similarity of central system and peripheral tumor microenvironments at the time of tumor occurrence, and the attempts to further consider the pan-tumor reference value of our study, a common tumor type outside the center system, LUAD (Lung adenocarcinoma), was selected. All the data verified that our results have certain implications for tumors both in and outside the central system, and strengthened the persuasive power of our data. Therefore, we believe that *FKBP5* can indicate the staging of PCNSL to some extent. Although more work is needed to validate the marker of organizers, the current results suggest that targeting this subgroup may inhibit the formation of a barrier environment, thereby improving immunotherapy.

### Molecular mechanism of the TME remodeling pattern

**Fig. 6:**
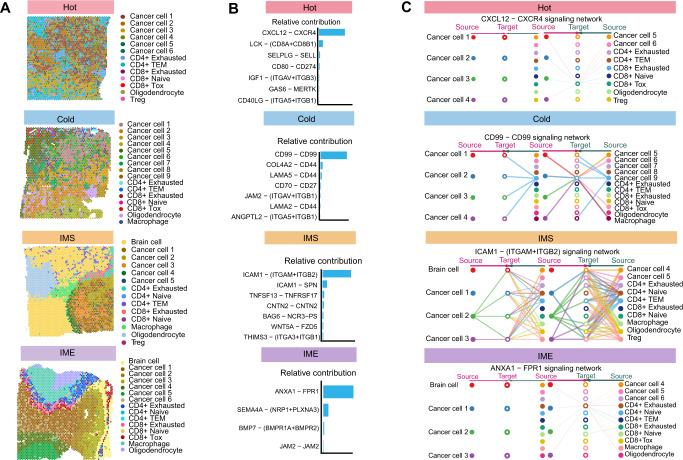
Molecular mechanism of the TME remodeling pattern. **A** Spatial distribution of immune cells and tumor cell subsets in each TME. **B** Relative contribution of receptor ligands in each TME. **C** Intercellular communication targets of each TME. See also Supplementary Fig. S7–S11.

Immune cells in the TME are also a population of interest, among which the status of T cells is particularly important. By calculating the gene expression profiles of each T-cell subset in the single-cell data, and then scoring the spot of each T-cell in the spatial transcriptome, the spatial representation of each type of T-cell was obtained. In hot tumors, we found that CD8 TOX T cells communicate most closely with other cells through “Cell Chat Analysis” (Fig. 6A and Supplementary Fig. S7A). Among all significant pathways in the whole communication network, the *CXCR4-CXCL12* signaling axis plays the most important role in hot tumors (Fig. 6B and Supplementary Fig. S8A, B). It can be seen from the analysis of incoming and outgoing signal patterns that CD8 TOX T cells are the most important signal receivers (Fig. 6C). The spatial expression patterns showed that *CXCR4-CXCL12* signaling axis was extensively activated in the hot TME (Supplementary Fig. S7B). In cold tumors, the main part of the cell communication network is the tumor cells of various subgroups (Supplementary Fig. S7C). The most important signaling axis in cold tumors was the *CD99* signaling axis (Fig. 6B and Supplementary Fig. S9A, B), and the population receiving this signal was the tumor cell subgroup with the strongest proliferative ability in the late development of cold tumors (Fig. 6C). In terms of the spatial expression pattern, the *CD99* signaling axis was widely distributed throughout the whole cold TME (Supplementary Fig. S7D). Communication between each cell population of IMS tumors is relatively intensive, and it is also a TME with the most kinds of signaling pathways, which forms a strong echo with the transition state of IMS tumors (Fig. 6A and Supplementary Fig. S7E). Among the numerous pathways, the *ITGAM* + *ITGB2-ICAM1* signaling axis had the most important significance (Fig. 6B and Supplementary Fig. S10A, B). The final recipients of this signaling axis are both immune cells and tumor cells, symbolizing the drastic change process of tumor cells and immune cells in this state (Fig. 6C). At the same time, the spatial distribution of the *ITGAM* + *ITGB2-ICAM1* signaling axis was also concentrated in the area of close contact between tumor cells and T cells, which further reflects the intense immune struggle in this area (Supplementary Fig. S7F). For IME tumors, the center of the signal network was CD4 exhausted T cells, and the intercellular interactions in the whole TME were much weaker than those in other TMEs (Fig. 6A and Supplementary Fig. S7G). Of only four pathways, the *ANXA1 - FPR1* signaling axis was the most important, and building a barrier environment and strengthening the signaling axes of tight connections took a secondary position (Fig. 6B and Supplementary Fig. S11A, B). The *ANXA1-FPR1* signaling axis works on CD4- and CD8-exhausted T cells, causing them to be in an anti-inflammatory state (Fig. 6C and Supplementary Fig. S7H). In addition, the barrier environment and the signaling axis that strengthen the tight connections between cells make it difficult for immune cells to enter the tumor. By observing the status of T cells in each TME, we noted that exhausted T cells were the majority of T cells clustered at invasive margins, suggesting that various molecules promoting exhaustion of T cells may play a crucial role, which is very important for tumor immunotherapy.

### Immunotherapy innovation for the TME remodeling pattern

**Fig. 7:**
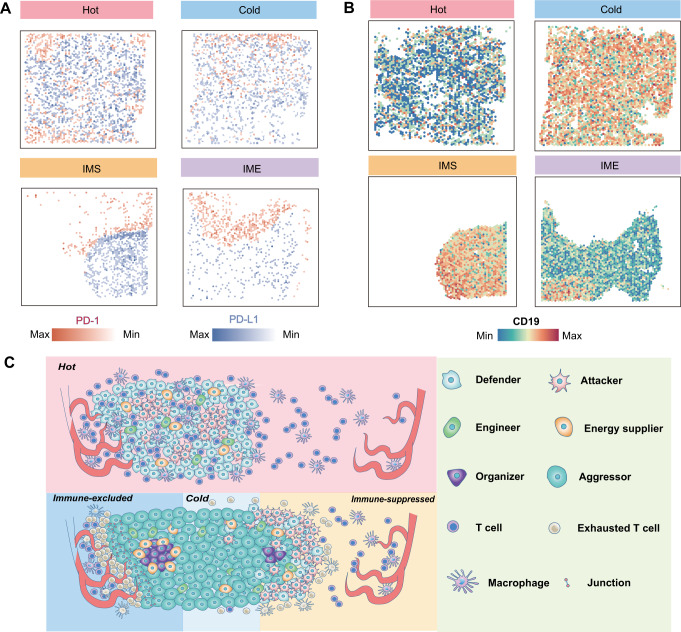
Immunotherapy innovation for the TME remodeling pattern. **A** Spatial feature plots of *PD-1*(red) and *PD-L1*(blue) expression in each TME. **B** Spatial feature plots of *CD19* expression in each TME. **C** The pattern diagram of TME remodeling pattern in PCNSL. See also Supplementary Fig. S12.

Immunotherapy targeting the TME has always been a key concern in this research field, and our research ultimately aims to provide new ideas and suggestions for tumor treatment. Among the current tumor immunotherapies, the use of immune checkpoint molecular inhibitors and CAR-T therapy have been proven to be the most effective [18–20]. First, we focused on to the spatial expression of immune checkpoint molecules and their corresponding ligands. It was found that the expression of immune checkpoint molecules and their receptors, such as PD-1 and PD-L1, could show obvious temporal and spatial heterogeneity in different TMEs. In hot environments, the expression of *PD-L1* was distributed throughout the TME and showed a trend of increased expression at the end of this developmental stage (Fig. 7A and Supplementary Fig. S12B). In the IMS stage of intense struggle between tumor cells and immune cells, *PD-L1* expression was concentrated at the invasive margin of tumor cells and immune cells, where it fully binds to PD-1 and promotes the exhaustion of T cells. However, the cells inside the tumor expressed less *PD-L1*. In cold and IME environments, tumor cells seemed to have become dominant and gradually decreased *PD-L1* expression compared to the IMS environment. We had also checked other immune checkpoint molecules and did not find any temporal or spatial distribution characteristics similar to those of *PD-L1* (Supplemental Fig. S12A).

In the treatment of CAR-T therapy, the main target, CD19, also has unique spatial expression differences. Hot tumors have relatively weak expression of *CD19* in tumor cells, but in cold and IMS tumors, the expression of *CD19* is significantly increased. In IME tumor, tumor cells with high expression of *CD19* are even encapsulated inside the tumor, making it difficult for CAR-T cells to play a targeting role, which is likely to be one of the reasons for the greatly reduced therapeutic effect (Fig. 7B). Interestingly, the expression of *CD19* showed a similar pattern to that of organizers in both spatial location and expression quantity, which showed that intervention with organizers might have a positive effect on immunotherapy from another perspective (Supplemental Fig. S12C). Our results showed that the expression of molecules in immunotherapy may not simply be an order of magnitude change or a dynamic change over time, their expression is likely to be accompanied by unique changes in spatial location. If we can take advantage of the unique expression mode of such molecules, it will be more conducive to improving the accuracy and efficiency of immunotherapy. Thus, the TME remodeling pattern of PCNSL and the corresponding key molecules and mechanisms were revealed (Fig. 7C).

## Discussion

Here, we presented the overall landscape and various information of the TME in a more complete manner through the combined use of single-cell and spatial transcriptome technologies, which not only ensures high resolution but also maintains the complete statues of the TME and restores the spatial location information of various PCNSL cells. Currently, studies on PCNSL are mainly clinically related. There are few studies on the TME of PCNSL, one of them described the cell types and compared lymphoma outside the CNS with PCNSL [12]. However, the data did not contain any spatial information. Another study focused on using single-cell transcriptome technology to describe the heterogeneity of malignant B cells in PCNSL and the corresponding immune cell state in detail, and used spatial transcriptome to show the overall spatial distribution of malignant B cells in different patients [21]. Our study combined four typical tumor microenvironment characteristics to explore the spatial heterogeneity of tumor cells in the PCNSL microenvironment, and further defined them according to their spatial localization and function, innovatively classifying tumor subpopulations through functional analysis as defenders, attackers, aggressors, organizers, engineers and energy suppliers.

Earlier studies have long assumed that the brain is an immune-privileged region [22, 23]. After the concept of CNS immunity was partially redefined, studies have demonstrated that blood-brain barrier permeability increases after tumor occurrence, and the TME in the brain has cell types similar to those outside the brain, except for the cells in the brain [13]. This new concept indicates that research on the TME of the CNS is also of reference value for other TMEs. On the other hand, the degree of immune cell infiltration in the TME is correlated with the immune situation in the region before TME formation [5]. Organs or tissues that are more susceptible to immune cell infiltration are more likely to push the TME to a hot environment or conversely to a cold environment. However, there are few immune cells in the brain before TME formation, making the TME, like PCNSL, have a relatively clean background before the occurrence of tumors. This kind of model may have certain advantages in the study of the TME.

Through spatial pseudo time series analysis, we found that tumor cells evolve through the TME remodeling pattern and filled the gap in the field of unclear tumor staging in PCNSL. In previous studies, the connections among the 4 kinds of TMEs were unclear, and the TMEs were classified based on the degree of infiltrating T cells [24–26]. Our study revealed the developmental sequence and functional correlation of each tumor subgroup. In contrast to previous studies [27, 28], we found that IMS is a transitional state of tumor progression after the hot state, while IME, which was previously considered to be a transitional state, is a terminal state of tumor progression similar to the cold state, but it belongs to a different cell fate. We also found that tumor cell fate selection is likely based on T-cell load, which we call the immune pressure-sensing model. When there are few T cells with immune killing ability in the TME, the tumor often chooses to enter the cold state and carry out strong proliferation and expansion. However, when T cells are heavily loaded, such as in areas close to blood vessels, where a large number of T cells may exist for backup, tumors often choose to enter the IME state in such areas and form a tight junction that isolates immune cells from the TME. Previous studies tended to provide their own treatment plans for individual TMEs [23, 29, 30], and the specific targets in each microenvironment were mostly screened based on a single microenvironment [31, 32]. However, they failed to take into full consideration that different microenvironments may coexist and have time and space continuity with each other. Our study is the first to integrate four major microenvironments on the same level and timeline and to take full account of the connections between different microenvironments. On this basis, we screened out the key molecules of tumor transition from the hot state to the two terminal states and provided more meaningful reference targets.

Significant progress has been made in the treatment of PCNSL, methotrexate-based multidrug chemotherapy is widely regarded as the standard of pretreatment, but the optimal treatment regimen for salvage treatment has not been determined [33, 34]. Several new treatment strategies are being tested, such as ibrutinib inhibitors, immunomodulatory drugs, PD-1 antibody and CAR-T cells [35–37]. Unfortunately, the beneficial effects of these novel drugs are often not long-lasting, and new treatment strategies that are more precise and durable are being developed. Our study provides some reasonable suggestions for current PCNSL therapy. The first, and most noteworthy, is to break down the barriers that tumor cells have built up. In the IME state, previous reports have found that breaking the tight intercellular connections and barrier environment can better inhibit tumor progression [38, 39]. Our study not only re-emphasizes the importance of breaking down this barrier environment but also identifies the mechanisms and key cell subsets, such as the FKBP5^+^ tumor subgroup, that contribute to the barrier effect. This will benefit future studies aiming to break down tumor walls in a more comprehensive and precise way and provides a possible method of tumor staging based on PCNSL pathological data, which is extremely important for the judgment and treatment of PCNSL patients.

CAR-T treatment of PCNSL may also face the problem of removing the barrier. CD19-targeted CAR-T cells have been successfully used to treat B-cell leukemia and lymphoma [40–42], however, their potential in PCNSL has not been fully explored, partly due to complications associated with treatment and partly due to specific and complex expression patterns of target molecules in the brain microenvironment [43]. Our study found that the expression pattern of *CD19* in PCNSL has unique characteristics, and CD19 is wrapped layer by layer in the interior of the barrier environment of IME, which also suggests that it is necessary to consider the removal of the barrier environment in the design of CAR molecules. One of the most important limitations in CAR-T treatment is the resistance of the CAR structure, some studies of other cancers showed that tumor cells decrease the expression of *CD19* to escape the CAR-T cells [44–46]. Our study shows that in PCNSL, *CD19* seems to have higher expression at the end of development than in the hot state at the earlier stage, and tumor cells take advantage of the barrier environment to avoid the attack of CAR-T cells. In the cold state, we found that the tumor *CD99* pathway plays a strong role, and it has been shown to be a potential new target for the CAR-T therapy of T-cell acute lymphoblastic leukemia [47]. Our results prove the validity of *CD99* in PCNSL, and it may serve as a potential target to inhibit the proliferation and migration of tumor cells at this stage.

Regarding the use of immune checkpoint molecule inhibitors, previous studies of PD-1 antibody therapy for PCNSL showed a response [48–50]. In our analysis of tumor heterogeneity in the TME, we found that tumor subgroups have their own spatial functional zones, which may not be considered in previous tumor treatments. Immune checkpoint molecule inhibitors have been proven to be potential drugs for PCNSL treatment. However, existing studies only focused on the expression levels of each immune checkpoint molecule in PCNSL patients [49, 51, 52], without paying attention to the dynamic change process over time or their spatial distribution. Our study found that the interaction of *PD-L1* and *PD-1* in the hot to IMS phases shifts the peak, mainly in the area of close contact between T cells and tumor cells, and after this period, *PD-L1* no longer has a major impact, therefore, in the immunotherapy of PCNSL, the medication time of a part of immune checkpoint molecule inhibitors is particularly important.

In conclusion, the uneven spatial distribution of tumor subsets and the uneven spatial distribution of immunotherapy-related target molecules may be responsible for poor therapeutic effects. In the selection of inhibitors and immune checkpoint drugs, TMEs in different states may have completely different responses to drugs, and the therapeutic effects of CAR-T therapy may also be different due to the spatial differences in TMEs. Our study details the characteristics of various types of TMEs and provides specific recommendations for drug selection and CAR molecular design. In addition, the developmental stages of different TMEs are clearly defined, and corresponding drugs and treatment methods can be selected and adjusted according to the developmental stages of tumors, which is of great significance in the precision treatment of tumors. Beyond these findings, our results further clarified that the spatial heterogeneity of tumors should be taken into consideration in immunotherapy. We expect that the collection of these data will provide valuable resources and meaningful references for future TME research, the development of antitumor drugs and the selection of treatment plans.

## Supplementary information

Supplementary Table 1

Supplementary Table 2

Supplementary Table 3

Supplementary Table 4

Supplementary Table 5

Supplementary Table 6

SUPPLEMENTAL INFORMATION

Supplementary Figure 1

Supplementary Figure 2

Supplementary Figure 3

Supplementary Figure 4

Supplementary Figure 5

Supplementary Figure 6

Supplementary Figure 7

Supplementary Figure 8

Supplementary Figure 9

Supplementary Figure 10

Supplementary Figure 11

Supplementary Figure 12

## Data Availability

Single cell sequencing data of PCNSL were obtained from Gene Expression Omnibus (GEO): GSE181304. Spatial transcriptome data of PCNSL were obtained from GSE230207. All analysis scripts and raw sequencing data reported in this paper are available upon reasonable request.
