# Supplementary material for: Spatial single cell analysis of tumor microenvironment remodeling pattern in primary central nervous system lymphoma
Source: Leukemia. 2023 Apr 29;37(7):1499–510. doi: 10.1038/s41375-023-01908-x (PMC10317840; doi:10.1038/s41375-023-01908-x)
Supplement: Supplementary file 7 — SUPPLEMENTAL INFORMATION [file 41375_2023_1908_MOESM7_ESM.docx]

**SUPPLEMENTAL INFORMATION
Supplemental Figure 1. Complete TMEs for each sample and marker genes of each cell type in TME.**(A) CD3 immunohistochemical staining of complete TMEs for each sample. The dotted line indicates the invasive margin of the tumor. Scale bar, 0.5 cm.
(B) Dotplot of the top marker genes in each cell cluster in scRNA-seq data.
(C) Dotplot of the top marker genes in each cell cluster of T-cell subsets.
 **Supplemental Figure 2. Scores of each cell type and the spatial expression of tumor cells and T cells.**(A) Violin plots of macrophage, microglial cell, oligodendrocyte T cell and cancer cell scores in each TME from each cluster of the scRNA-seq data. The cluster with the highest average score is shown in the red dotted box.
(B) Spatial feature plots of tumor cell and T-cell markers in the “hot” TME.
(C) Spatial feature plots of tumor cell and T-cell scores in the “hot” TME.
(D) Spatial feature plots of tumor cell and T-cell markers in the “cold” TME.
(E) Spatial feature plots of tumor cell and T-cell scores in the “cold” TME.
(F) Spatial feature plots of tumor cell and T-cell markers in the “IMS” TME.
(G) Spatial feature plots of tumor cell and T-cell scores in the “IMS” TME.
(H) Spatial feature plots of tumor cell and T-cell markers in the “IME” TME.
(I) Spatial feature plots of tumor cell and T-cell scores in the “IME” TME.
 **Supplemental Figure 3. Functional classification of tumor cell subsets and evaluation of immune regulation status of four TME.**(A) Functional classification of tumor cell subsets of each TME.
(B) Evaluation of immune regulation status of each TME.
(C) The subpopulations of various role types of tumor cells in scRNA-seq data of PCNSL patients and top 5 GO term of each role types were shown around the corresponding subpopulations.

**Supplemental Figure 4. The developmental trajectory of tumor subsets in each TME.**
(A) The temporal and spatial developmental trajectories of various tumor cell subpopulations in the “hot” TME. The arrow indicates the direction of cancer cell development. The dots are the nodes that generate new subgroups during development, and the larger dot is the starting point of development.
(B) The temporal and spatial developmental trajectories of various tumor cell subpopulations in the “cold” TME.
(C) The temporal and spatial developmental trajectories of various tumor cell subpopulations in the “IMS” TME.
(D) The temporal and spatial developmental trajectories of various tumor cell subpopulations in the “IME” TME.
 **Supplemental Figure 5. Analysis of the key genes in the developmental trajectory.**(A) The key gene screening of developmental trajectory nodes.
(B) Gene Switch analysis of the “hot” to “cold” tumor development.
(C) Gene Switch analysis of the “hot” to “IME” tumor development.
(D) Hematoxylin and eosin (H&E) staining of the “IME” environment. The dotted boxes and arrows indicate the distribution of blood vessels. **Supplemental Figure 6. Weighted gene coexpression network analysis (WGCNA) of the “organizers”**(A) WGCNA of the “organizers” subpopulation. Two modules and 902 oligogenes were successfully clustered. Each branch of the tree represents genes, and genes in the same module have the same module color. The gray module summarizes the oligomeric genes.
(B) Gene set variation analysis (GSVA) of “organizers” -related pathways of 2 modules.

**Supplemental Figure 7. Communication relationships and spatial feature plots of key pathway receptor and ligand molecular expressions in each TME.**(A) Shell diagram of the communication relationships and weights of various cell subpopulations in the “hot” TME.
(B) Spatial feature plots of *CXCL12* and *CXCR4* expression in the “hot” TME.
(C) Shell diagram of the communication relationships and weights of various cell subpopulations in the “cold” TME.
(D) Spatial feature plots of *CD99* expression in the “cold” TME.
(E) Shell diagram of the communication relationships and weights of various cell subpopulations in the “IMS” TME.
(F) Spatial feature plots of the *ICAM1* and *ITGAM* expression in the “IMS” TME. The region inside the dotted line is the region where tumor cells are mainly distributed.
(G) Shell diagram of the communication relationships and weights of various cell subpopulations in the “IME” TME.
(H) Spatial feature plots of *ANXA1* and *FPR1* expression in the “IME” TME. The region inside the dotted line is the region where tumor cells are mainly distributed.
 **Supplemental Figure 8. Cell communication patterns in the “hot” TME.**(A) Dotplot of the major cellular communication modes in the “hot” TME.
(B) Chord chart of the major cellular communication modes in the “hot” TME. **Supplemental Figure 9. Cell communication patterns in the “cold” TME.**(A) Dotplot of the major cellular communication modes in the “cold” TME.
(B) Chord chart of the major cellular communication modes in the “cold” TME. **Supplemental Figure 10. Cell communication patterns in the “IMS” TME.**(A) Dotplot of the major cellular communication modes in the “IMS” TME.
(B) Chord chart of the major cellular communication modes in the “IMS” TME.

**Supplemental Figure 11. Cell communication patterns in the “IME” TME.**(A) Dotplot of the major cellular communication modes in the “IME” TME.
(B) Chord chart of the major cellular communication modes in the “IME” TME.

**Supplemental Figure 12. Spatial expression patterns of immune checkpoint molecules and CAR-T target molecule.**(A) Spatial feature plots of two pairs of immune checkpoint molecules (left: TNFRSF9 (red) and TNFSF9 (blue); right: IDO-1 (red) and AHR (blue)) expression in each TME.
(B) The temporal expression of *PD-L1* in tumor cells in each TME.
(C) Violin plots of *CD19* expression in each tumor subset. Clusters with a role defined as an “organizer” are shown in the red box.

**Supplemental Table 1.** Gene expression profile of each cell type in PCSNL single-cell data.

**Supplemental Table 2. Gene Ontology (GO) functions of each cluster of cancer cells.**

**Supplemental Table 3. The details of the definition and notation of PCNSL cell subpopulations.**
 **Supplemental Table 4. The top 100 hub genes in each module of WGCNA analysis.**
 **Supplemental Table 5. List of genes screened by LASSO regression.**

**Supplemental Table 6. List of genes screened by Cox multivariate regression.**
