# Supplementary figures and images for: Spatial single cell analysis of tumor microenvironment remodeling pattern in primary central nervous system lymphoma
Source: Leukemia. 2023 Apr 29;37(7):1499–510. doi: 10.1038/s41375-023-01908-x (PMC10317840; doi:10.1038/s41375-023-01908-x)

Figure S1

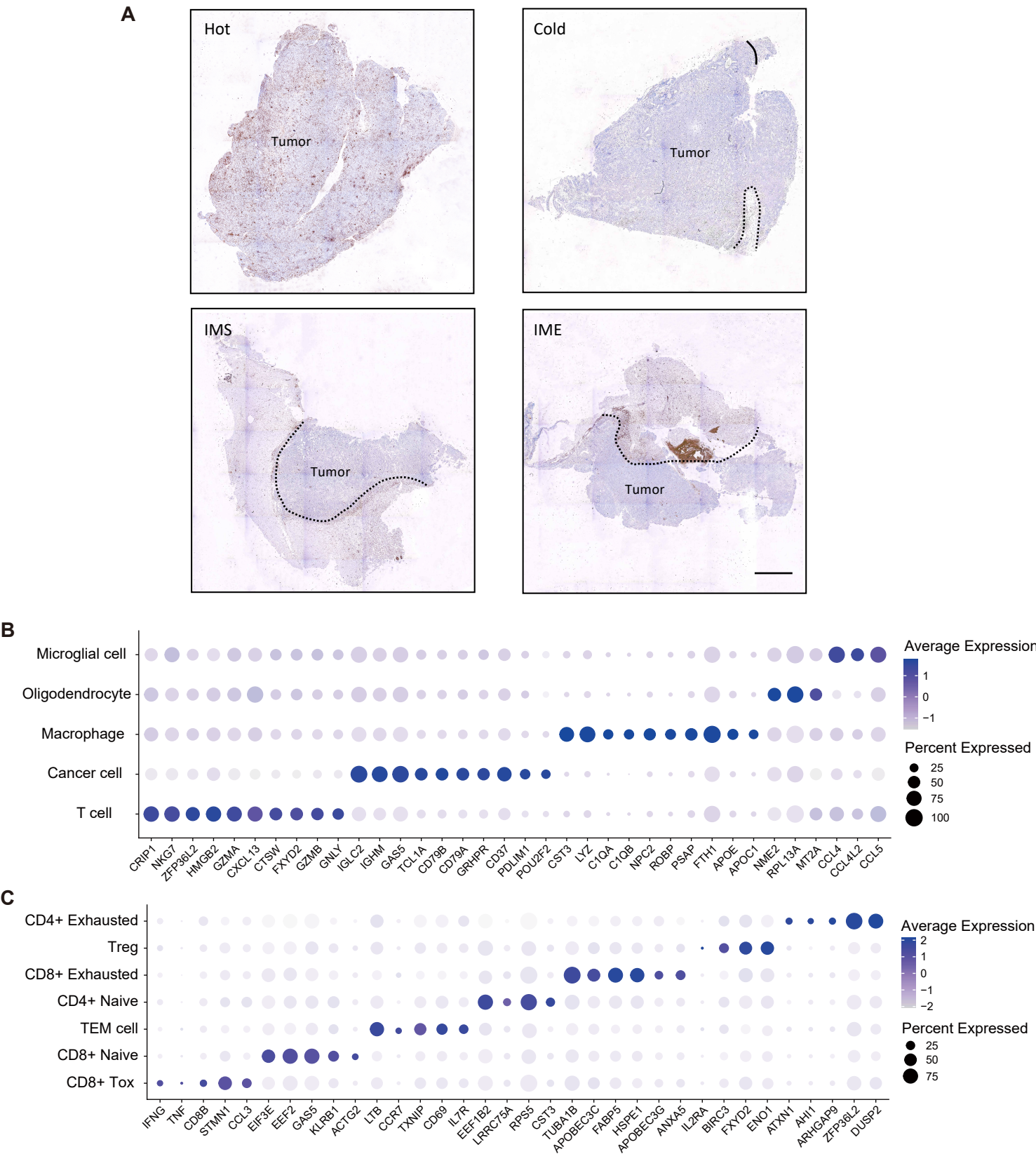

Supplement: Supplementary file 8 — Supplementary Figure 1 [file 41375_2023_1908_MOESM8_ESM.pdf]

Figure S2

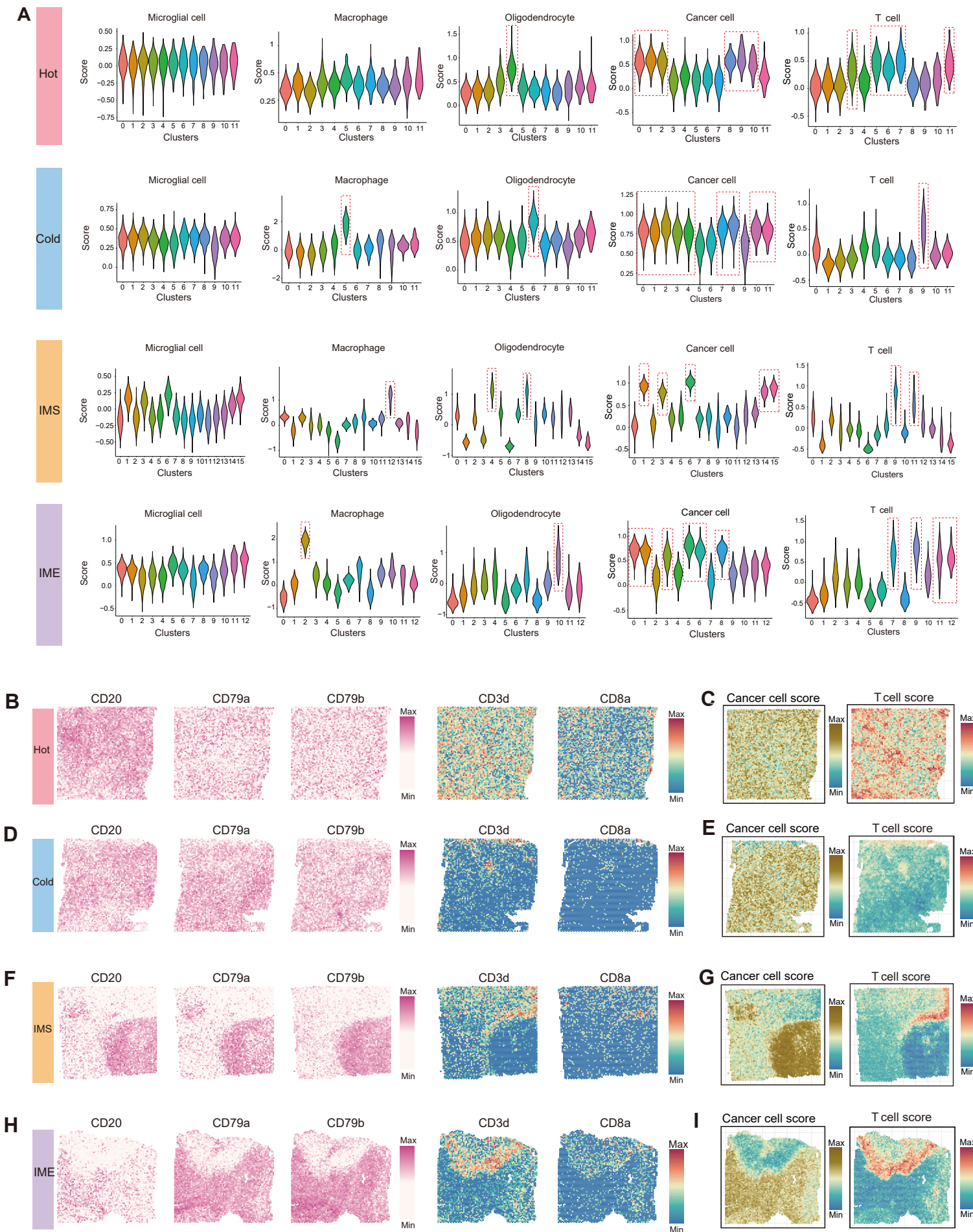

Supplement: Supplementary file 9 — Supplementary Figure 2 [file 41375_2023_1908_MOESM9_ESM.pdf]

**Figure S3**

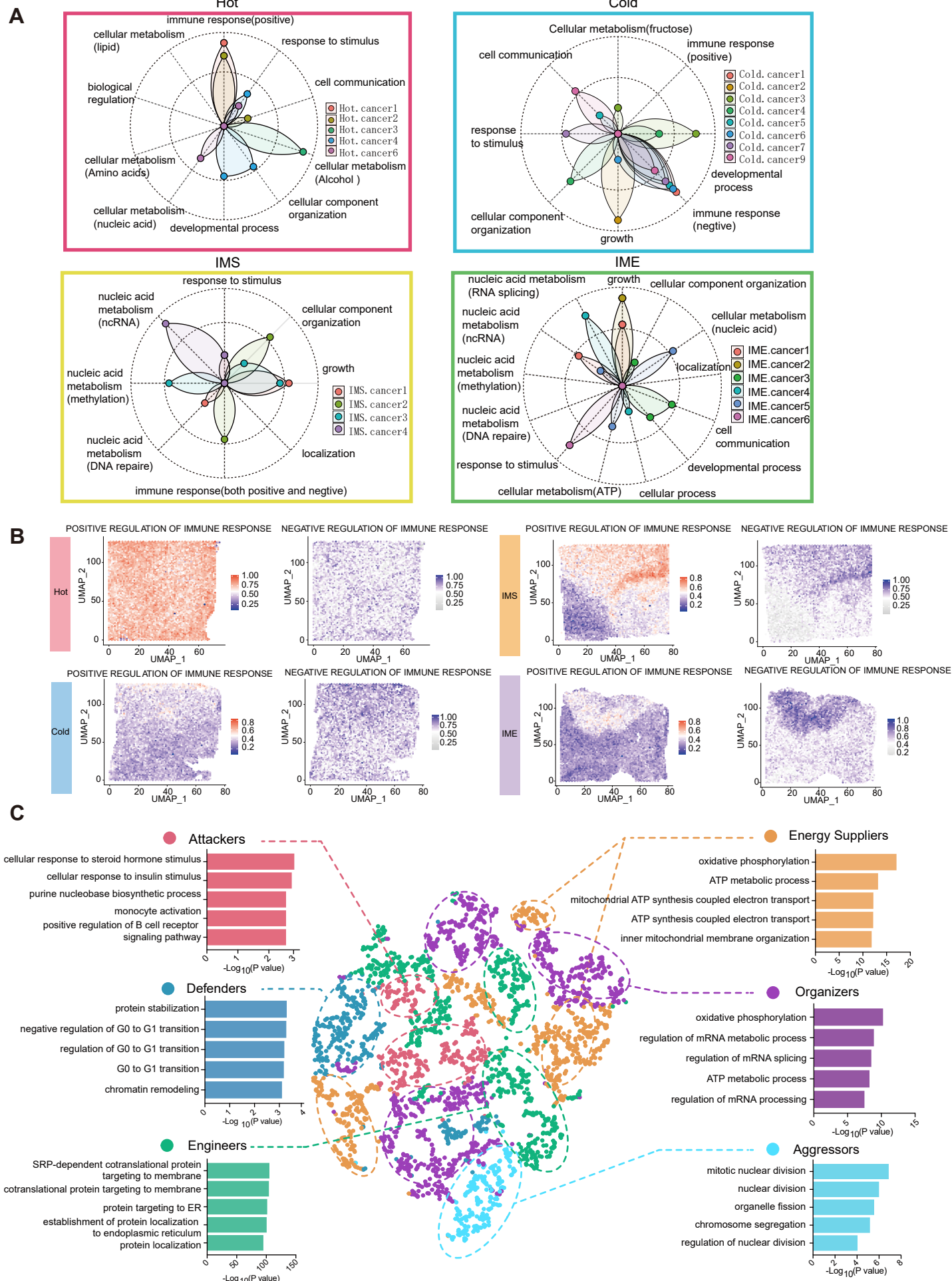

Supplement: Supplementary file 10 — Supplementary Figure 3 [file 41375_2023_1908_MOESM10_ESM.pdf]

Figure S4

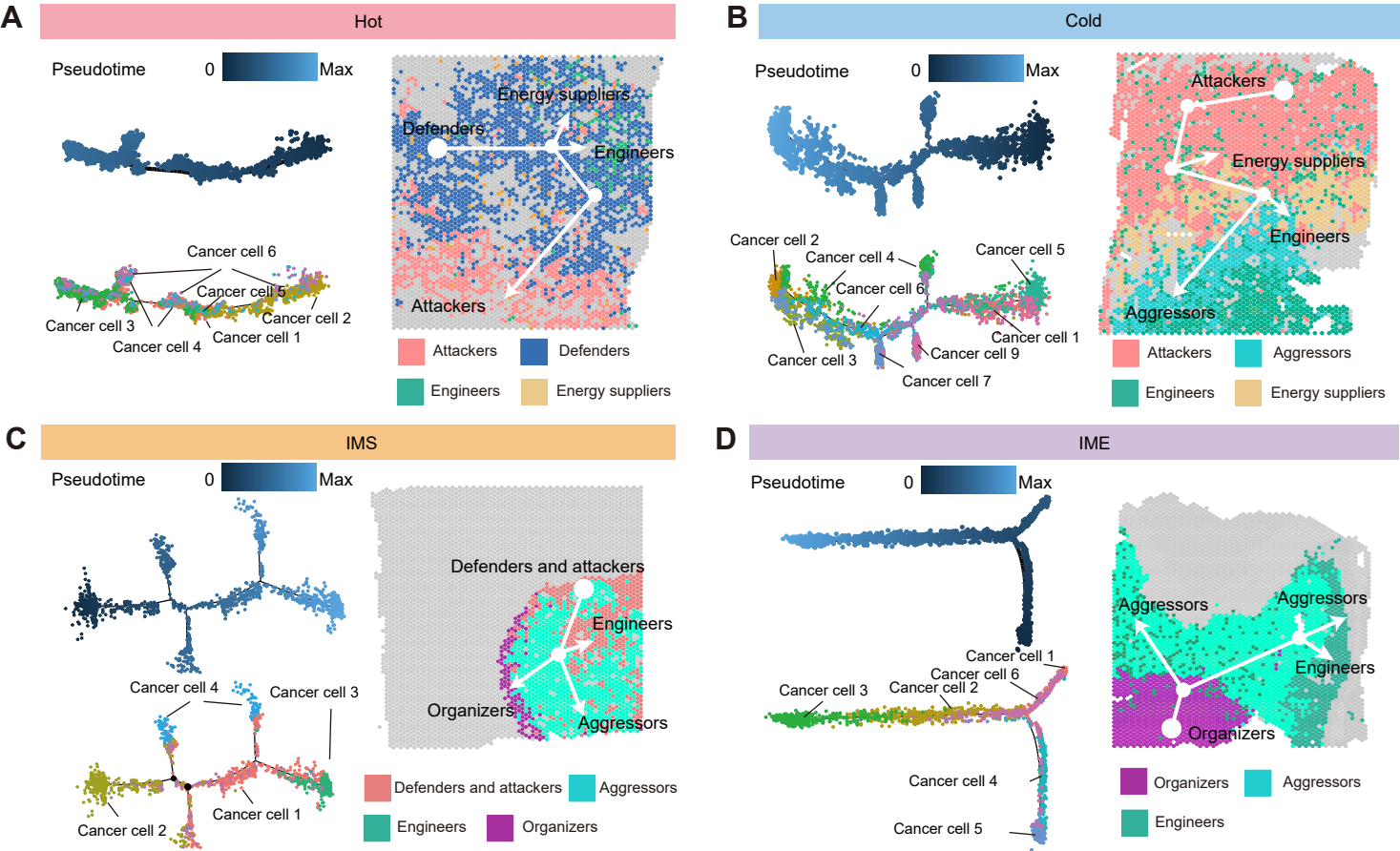

Supplement: Supplementary file 11 — Supplementary Figure 4 [file 41375_2023_1908_MOESM11_ESM.pdf]

Figure S5

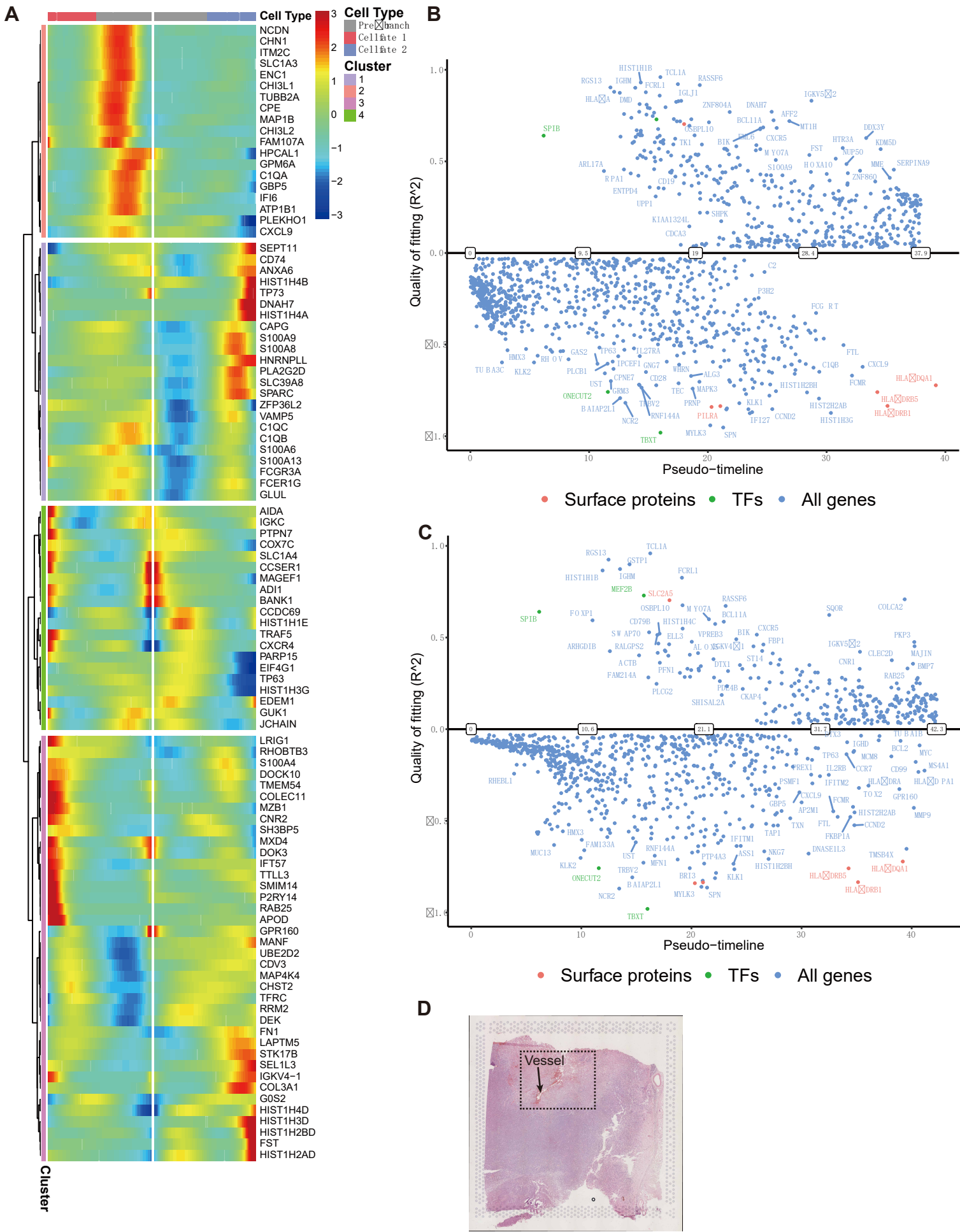

Supplement: Supplementary file 12 — Supplementary Figure 5 [file 41375_2023_1908_MOESM12_ESM.pdf]

Figure S6

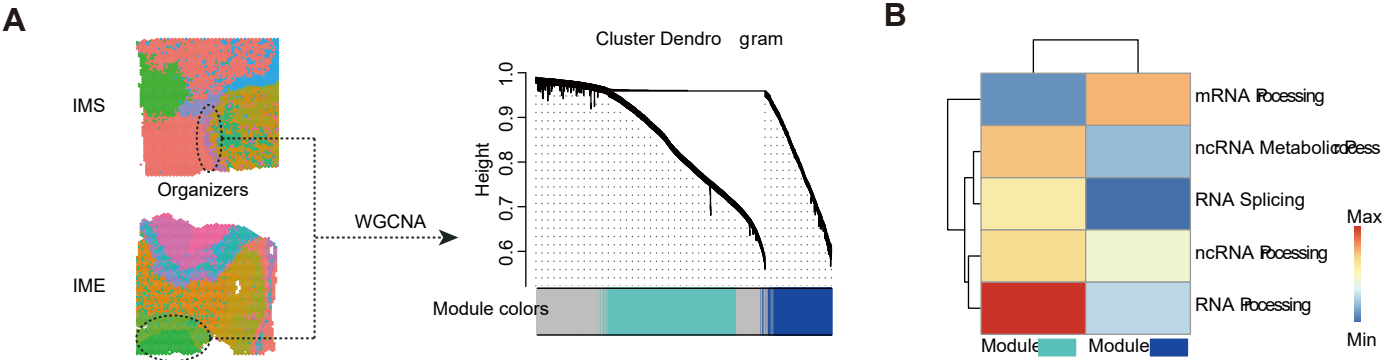

Supplement: Supplementary file 13 — Supplementary Figure 6 [file 41375_2023_1908_MOESM13_ESM.pdf]

Figure S7

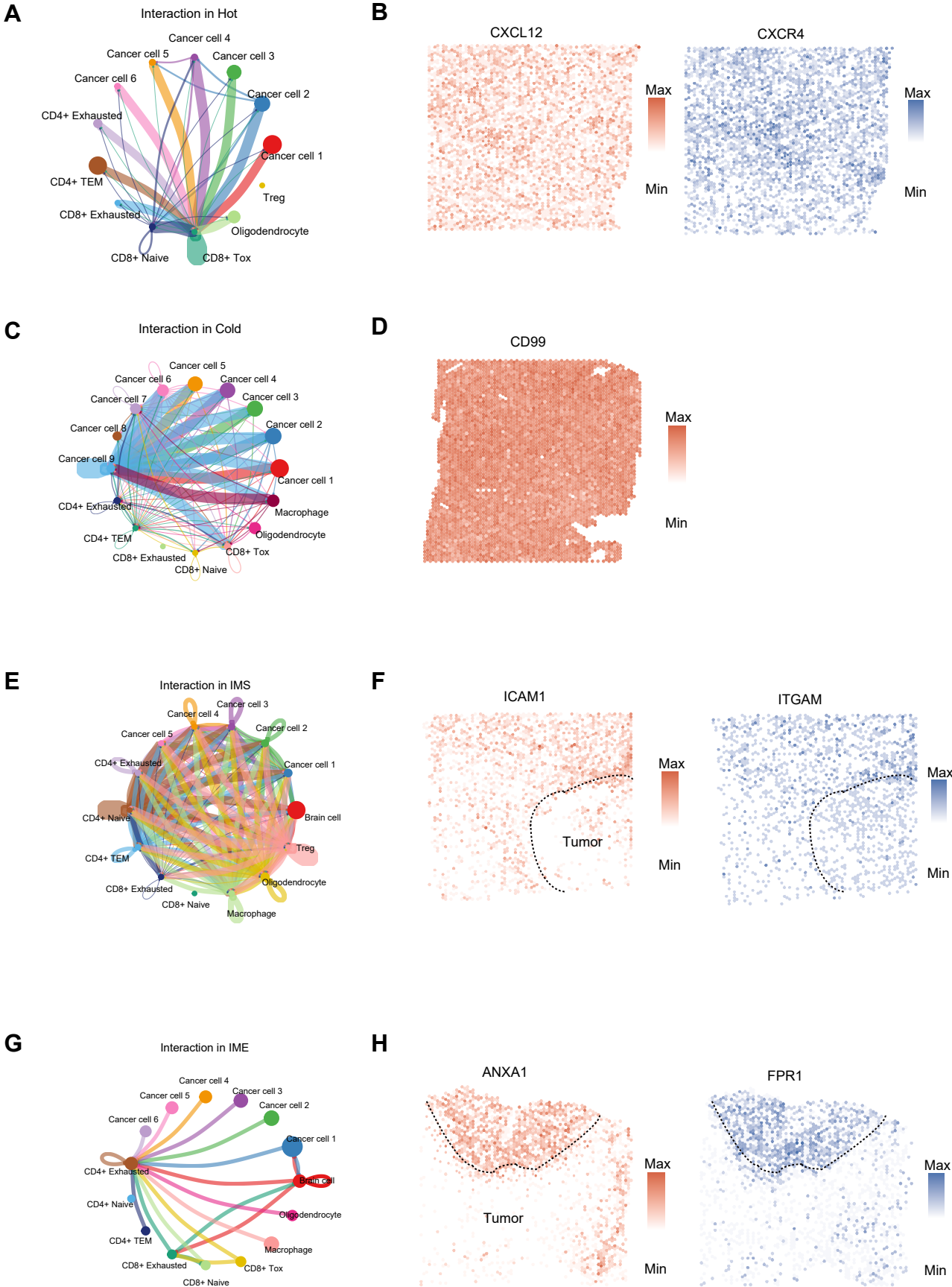

Supplement: Supplementary file 14 — Supplementary Figure 7 [file 41375_2023_1908_MOESM14_ESM.pdf]

**A**

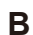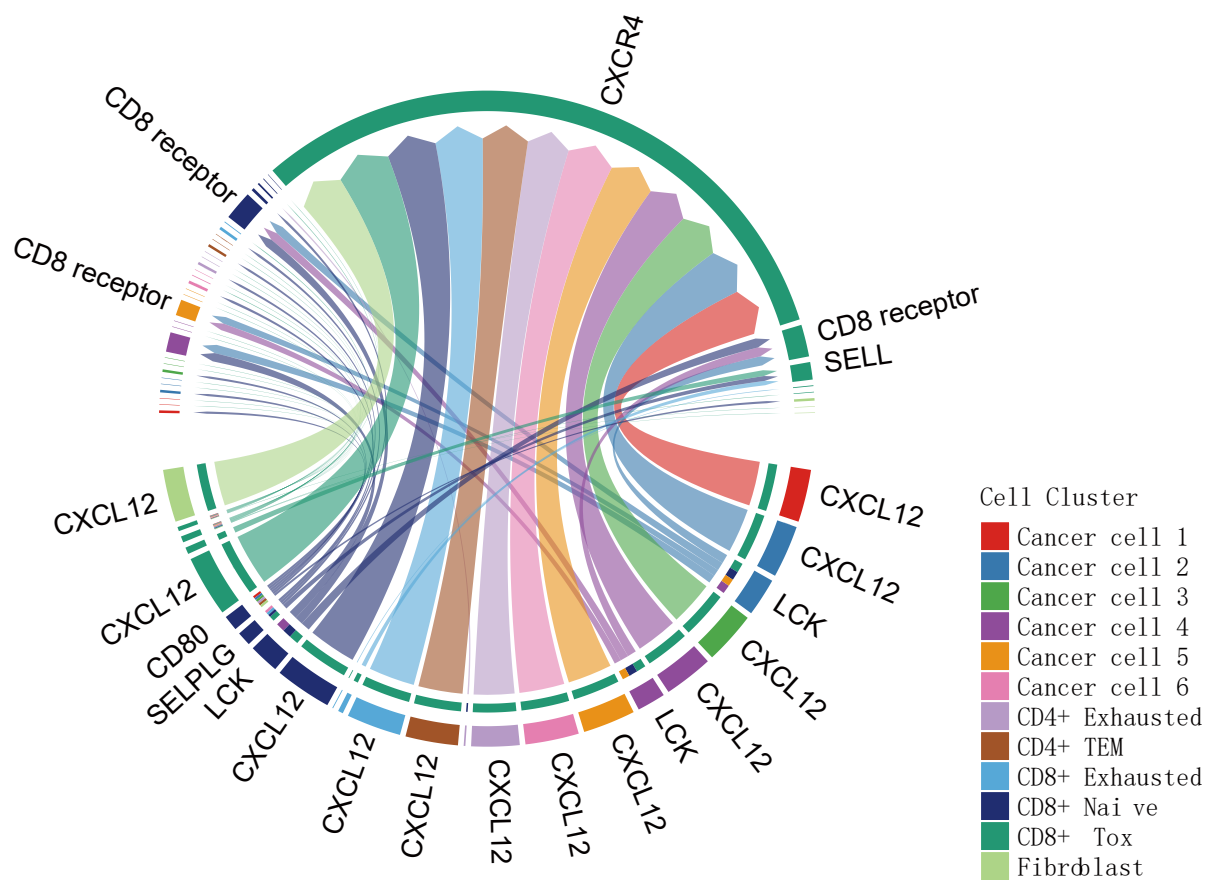

Supplement: Supplementary file 15 — Supplementary Figure 8 [file 41375_2023_1908_MOESM15_ESM.pdf]

**A**

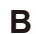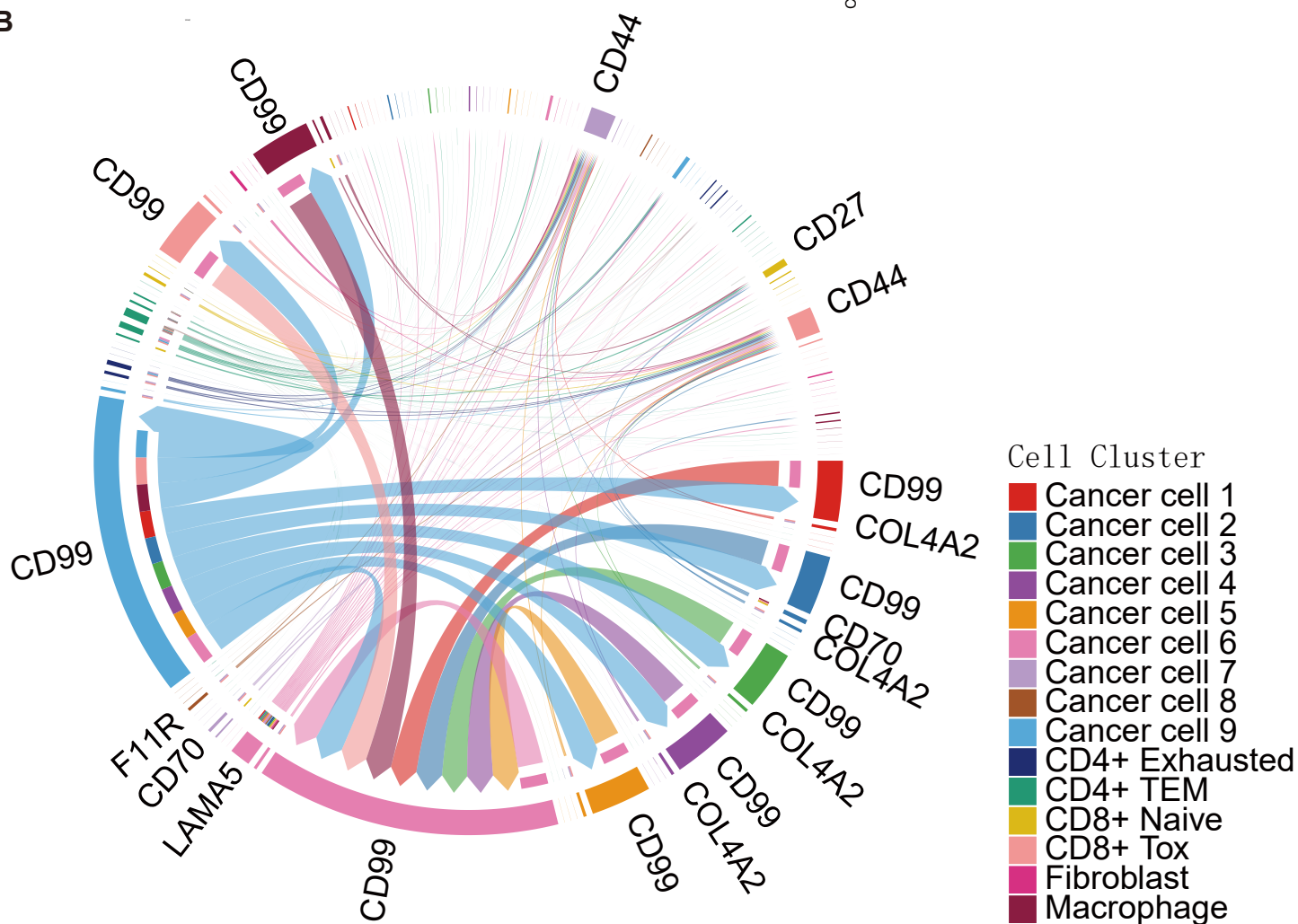

Supplement: Supplementary file 16 — Supplementary Figure 9 [file 41375_2023_1908_MOESM16_ESM.pdf]

**Figure S10**

**A**

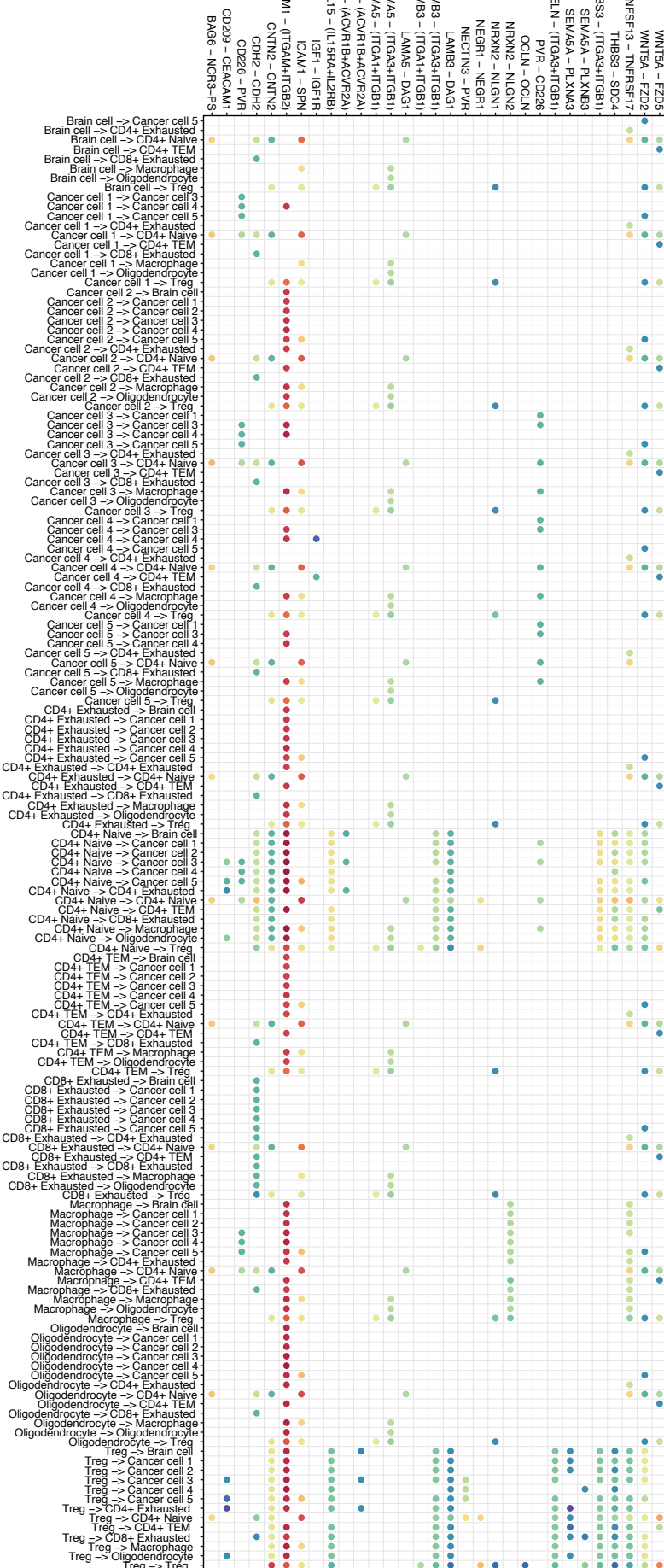

**B**

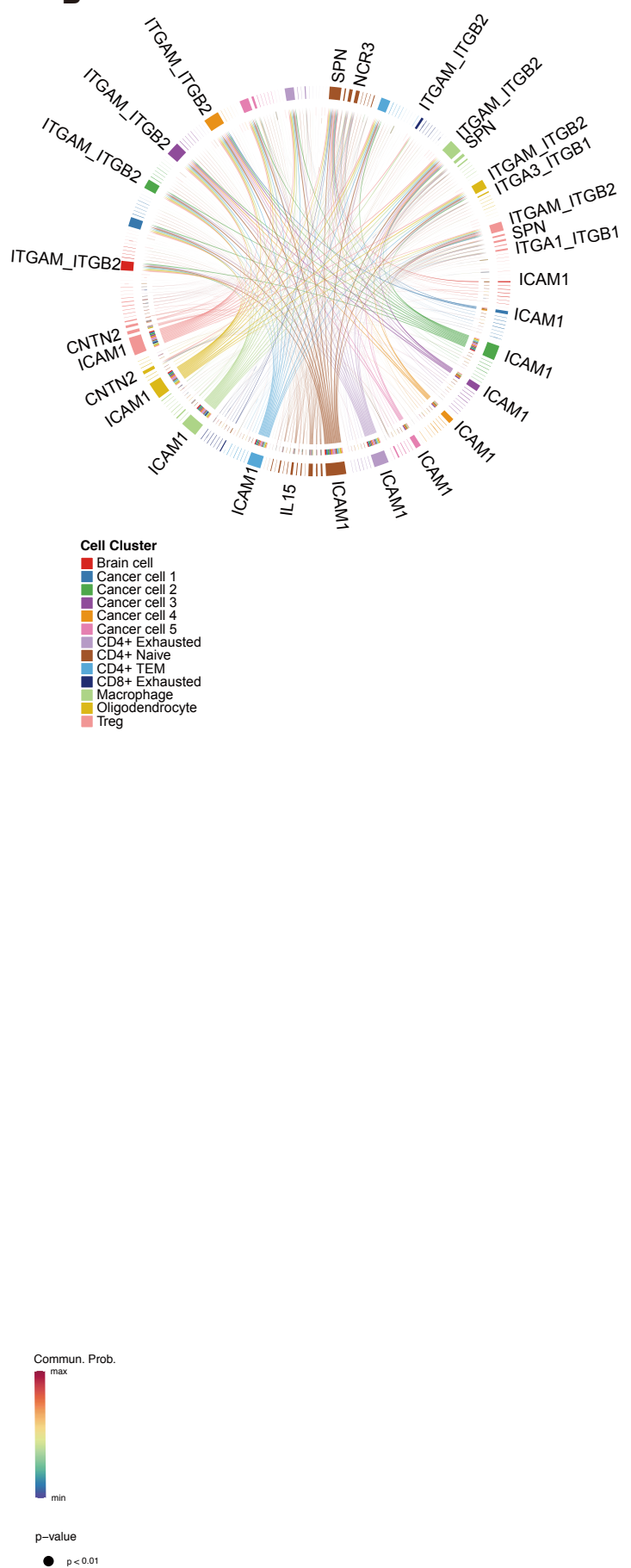

Supplement: Supplementary file 17 — Supplementary Figure 10 [file 41375_2023_1908_MOESM17_ESM.pdf]

**A**

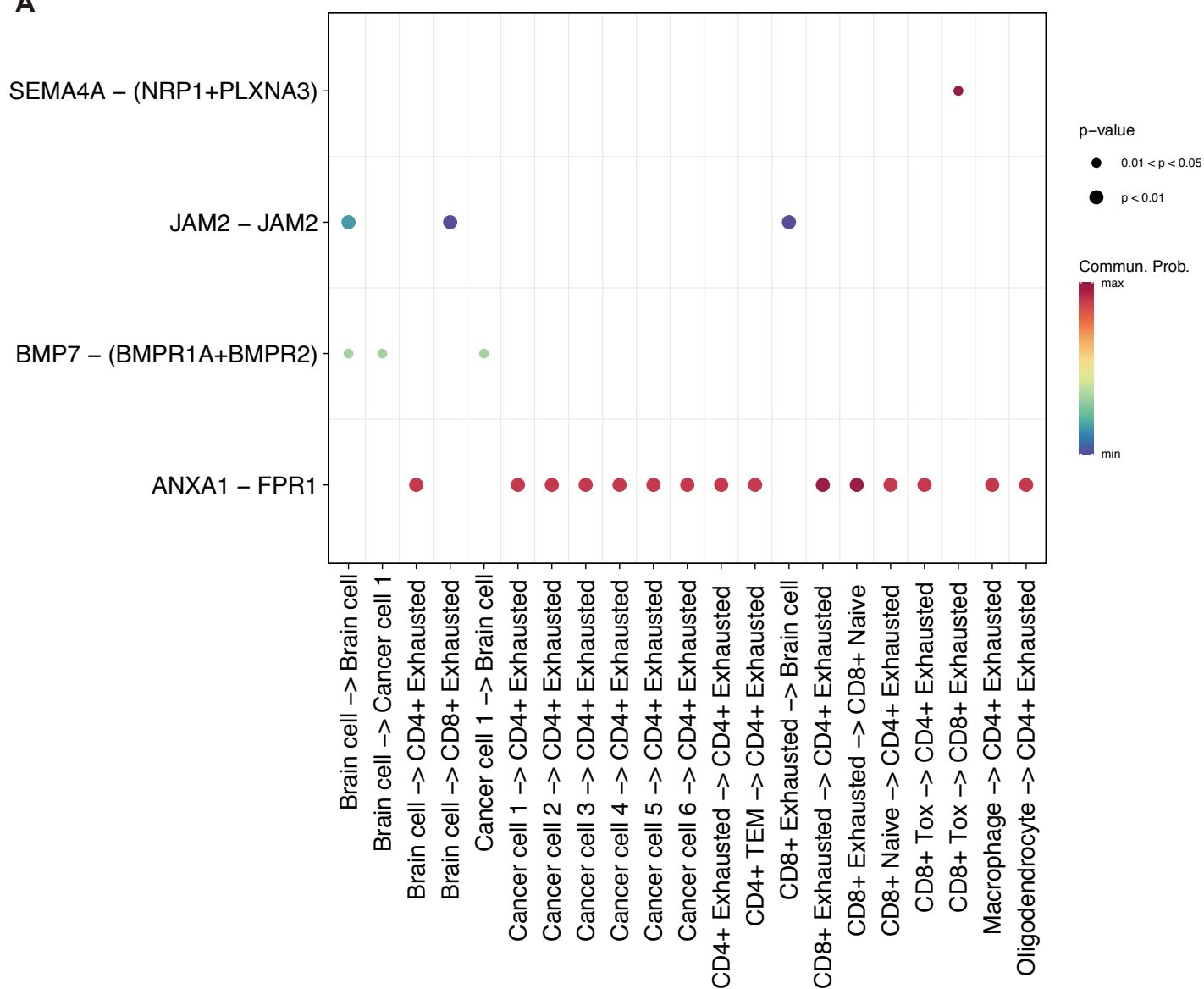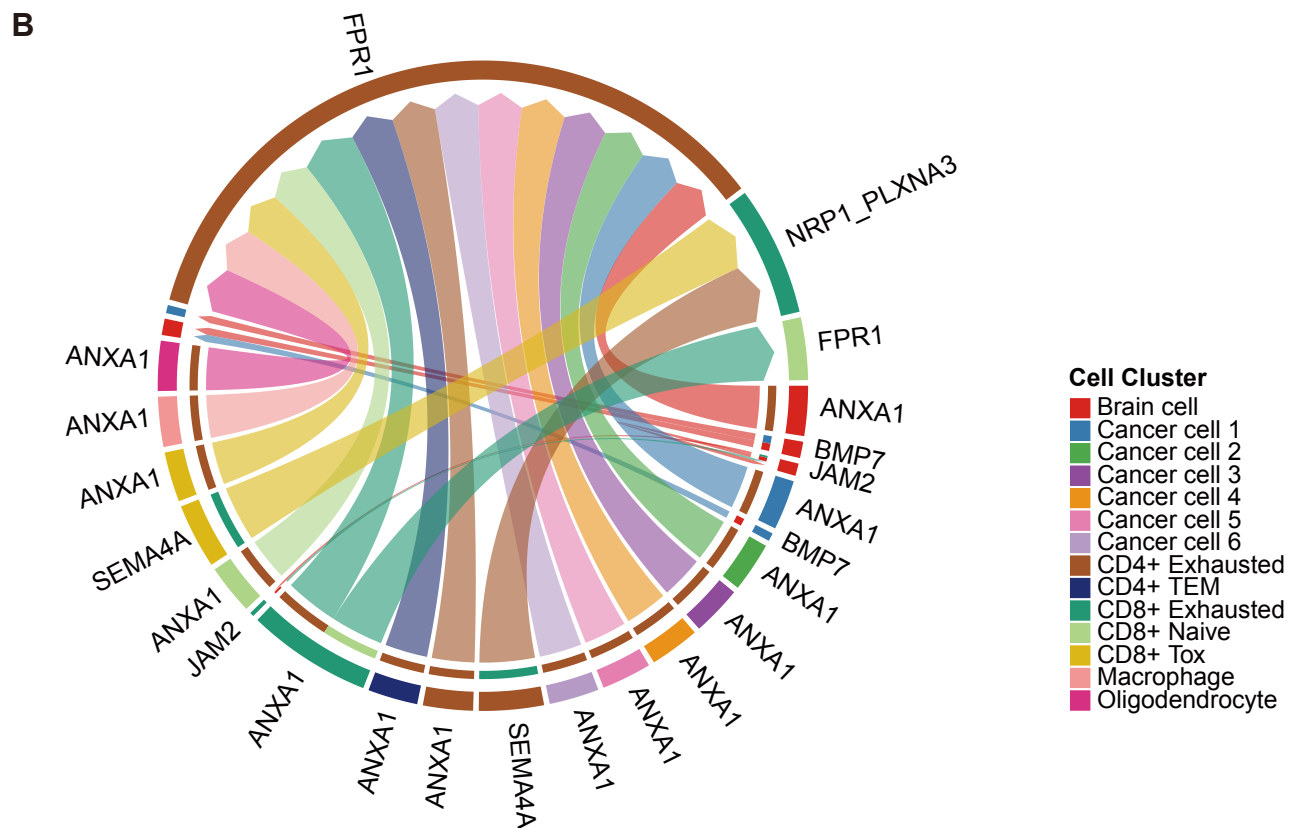

Supplement: Supplementary file 18 — Supplementary Figure 11 [file 41375_2023_1908_MOESM18_ESM.pdf]

### Figure S12

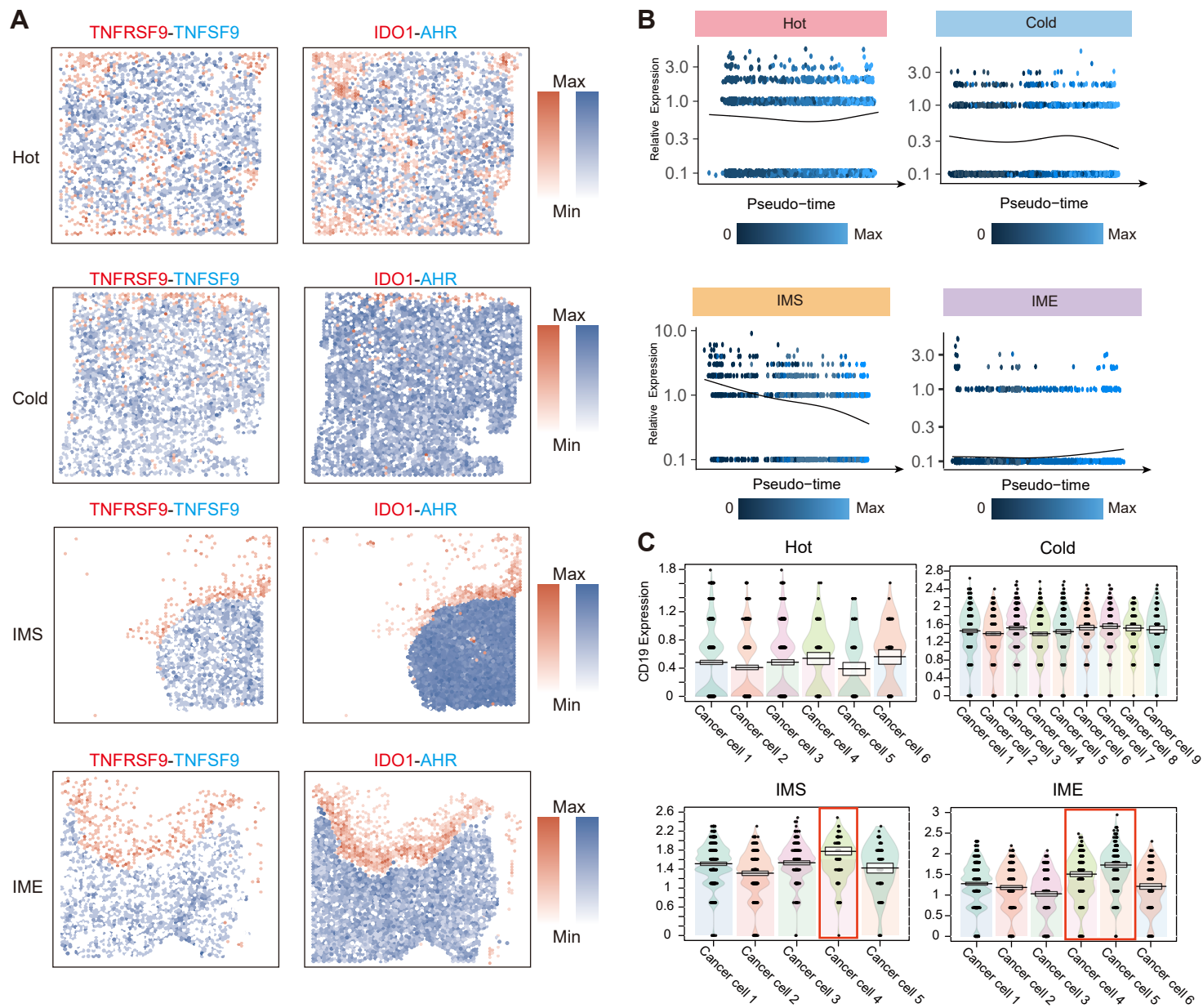

Supplement: Supplementary file 19 — Supplementary Figure 12 [file 41375_2023_1908_MOESM19_ESM.pdf]
